# The Role of Cancer Stem Cells in Colorectal Cancer: From the Basics to Novel Clinical Trials

**DOI:** 10.3390/cancers13051092

**Published:** 2021-03-04

**Authors:** Céline Hervieu, Niki Christou, Serge Battu, Muriel Mathonnet

**Affiliations:** 1 EA 3842 CAPTuR “Control of Cell Activation in Tumor Progression and Therapeutic Resistance”, Faculty of Medicine, Genomics, Environment, Immunity, Health and Therapeutics (GEIST) Institute, University of Limoges, 87025 Limoges CEDEX, France; celine.hervieu@unilim.fr (C.H.); christou.niki19@gmail.com (N.C.); serge.battu@unilim.fr (S.B.); 2 Department of General, Endocrine and Digestive Surgery, University Hospital of Limoges, 87025 Limoges CEDEX, France

**Keywords:** colorectal cancer, cancer stem cells, drug resistance, clinical trials

## Abstract

**Simple Summary:**

Cancer stem cells (CSCs) fuel tumor growth, metastasis and resistance to therapy in colorectal cancer (CRC). These cells therefore represent a promising target for the treatment of CRC but are difficult to study because of the complexity of their isolation. This review presents the methods currently used to isolate colorectal CSCs as well as the techniques for characterizing these cells with their advantages and limitations. The aim of this review is to provide a state-of-the-art on the clinical relevance of CSCs in CRC by outlining current treatments for CRC, the resistance mechanisms developed by CSCs to overcome them, and ongoing clinical trials of drugs targeting CSCs in CRC. Overall, this review addresses the complexity of studying CSCs in CRC research and developing clinically effective treatments to enable CRC patients to achieve a short and long-term therapeutic response.

**Abstract:**

The treatment options available for colorectal cancer (CRC) have increased over the years and have significantly improved the overall survival of CRC patients. However, the response rate for CRC patients with metastatic disease remains low and decreases with subsequent lines of therapy. The clinical management of patients with metastatic CRC (mCRC) presents a unique challenge in balancing the benefits and harms while considering disease progression, treatment-related toxicities, drug resistance and the patient’s overall quality of life. Despite the initial success of therapy, the development of drug resistance can lead to therapy failure and relapse in cancer patients, which can be attributed to the cancer stem cells (CSCs). Thus, colorectal CSCs (CCSCs) contribute to therapy resistance but also to tumor initiation and metastasis development, making them attractive potential targets for the treatment of CRC. This review presents the available CCSC isolation methods, the clinical relevance of these CCSCs, the mechanisms of drug resistance associated with CCSCs and the ongoing clinical trials targeting these CCSCs. Novel therapeutic strategies are needed to effectively eradicate both tumor growth and metastasis, while taking into account the tumor microenvironment (TME) which plays a key role in tumor cell plasticity.

## 1. Introduction

Colorectal cancer (CRC) is the fourth leading cause of cancer-related death worldwide [1]. While the occurrence and mortality rates of CRC is declining in the European countries, these rates are increasing in rapidly transitioning countries, such as many African and South Asian countries [2]. The tumor–node–metastases (TNM) classification allows the stratification of patient groups according to the stage of the disease, based on anatomical information [3,4]. The location and stage of the tumor enable both the assessment of the patient’s prognosis and the determination of the therapeutic approach, depending on the patient’s overall health as well as the status of the tumor in terms of mutation and mismatch repair (MMR) [1,5]. Therapeutic options for the treatment of CRC are surgical resection, systemic therapy including chemotherapy, targeted therapy and immunotherapy, local therapy for metastases and palliative therapy [1,6]. Importantly, surgical resection is the only curative treatment, if all macroscopic and microscopic tumor foci can be removed [1,6]. Unfortunately, even after well directed curative treatment, some patients experience treatment failure that may be associated with the development of multidrug resistance (MDR) during or after treatment. In addition, despite initially successful therapy, the development of drug resistance often leads to relapse in cancer patients, known as minimal residual disease (MRD) [7]. Both MDR and MRD can be attributed to a subpopulation of tumor cells with self-renewal and multi-lineage differentiation capabilities, the cancer stem cells (CSCs), known as colorectal cancer stem cells (CCSCs) for CRC [8]. CSCs contribute to tumor initiation and dissemination, treatment resistance and metastasis development. Tumor microenvironment (TME) and metabolic plasticity may also be involved in therapeutic failure by imposing selective pressures on cancer cells that lead to chemoresistance and cancer progression [9,10]. Therefore, the development of new therapies targeting CSCs, taking into account the TME and tumor metabolism, represents an interesting approach to overcome resistance to therapies [11]. In this review, we will present the origin of CCSCs and provide an overview of the techniques currently used to isolate them. Then, we will review current knowledge on the clinical relevance of CCSCs, through the clinical management of CRC and the mechanisms of resistance to therapies associated with CCSCs. Finally, we will introduce some clinical trials based on drugs targeting CCSCs.

## 2. Colorectal Cancer Stem Cells

The CSC theory suggests that tumor growth is driven by a small number of dedicated stem cells (SCs), the CSCs [8]. By definition, a CSC has the ability to self-renew in order to expand its pool and to generate all the differentiated cells that comprise the tumor (multi-potency). The transformation of a colorectal stem cell into CCSC requires the acquisition of tumor-related features.

### 2.1. Colorectal Cancer Stem Cell Origin

The history of CSCs began two decades ago with the discovery of CSCs in human acute myeloid leukemia (AML) by Dick and colleagues [12]. For the first time, a cell capable of initiating human AML in immunodeficient mice and possessing differentiation, proliferation and self-renewal capabilities was described. A few years later, using similar experimental approaches, the presence of CSC was demonstrated in solid cancers such as colorectal cancer. The origin of CSCs in CRC is controversial, and several hypotheses have been proposed. CCSCs are associated with the acquisition of malignant molecular and cellular changes either due to the accumulation of genetic and epigenetic alterations in restricted stem/progenitor cells and normal tumor cells, or to the dedifferentiation of somatic cells caused by various genetic and environmental factors [13,14,15]. CSCCs exhibit tumor-related characteristics such as uncontrolled growth, tumorigenicity and therapy resistance, and may constitute the small reservoir of drug-resistant cells that are responsible for relapses after chemotherapy-induced remission, known as MRD, and distant metastasis [7,11]. Thus, CCSCs play a key role in the initiation, invasion and progression of CRC as well as resistance to therapy. These CCSCs give rise to heterogeneous tumors that can be serially transplanted into immunodeficient mice that resemble the original tumor [16]. In addition, CCSCs have the ability to form disseminated metastatic tumors due to their extensive proliferative potential [15]. One of the main challenges in the study of CCSCs is their isolation, due to their low percentage within the tumor [16]. However, the CCSC population appears to be phenotypically and functionally heterogeneous and dynamic, which is another barrier to their isolation [17]. Therefore, the development of therapies that selectively eradicate CCSCs offers promising opportunities for a sustainable clinical response but requires effective technologies to detect and isolate them [11].

### 2.2. Colorectal Cancer Stem Cell Isolation Methods

Different methods are used to isolate CCSCs, based either on the expression pattern of CCSC markers, the functional aspect of CCSCs, or their biophysical features [18]. The objective of this chapter is to present the techniques currently in use with the advantages and disadvantages of each approach.

#### 2.2.1. CCSC Isolation Based on Phenotypic Features

Many stem cells markers were found to be associated with CCSC features. However, the heterogeneous and dynamic nature of CCSCs challenges their isolation and enrichment. The first publications from the literature identifying subpopulations of CSCs in CRC are summarized in Table 1. Experimental models, CCSC isolation methods and characterization techniques used by the authors are detailed in this table. Studies conducted by O’Brien et al. and Ricci-Vitiani et al. identified the first CCSC marker: the five-transmembrane glycoprotein CD133 [19,20]. However, its use has become controversial as the tumorigenic and clonogenic potential of CD133^+^-CSCs depends on the positivity for a specific glycosylated epitope of the CD133 protein [21].

Then, Clarke’s group showed that EpCAM^high^/CD44^+^cells isolated from human CRC could establish a tumor in mice with morphological and phenotypic heterogeneity of the original tumor and concluded that CD44 and EPCAM markers could be considered robust CCSC markers [22]. In addition, the study by Dalerba et al. highlights an additional differentially expressed marker, CD166, which could be used to further enrich CCSCs in the EpCA^high^/CD44^+^ population [22]. Using lineage-tracing experiments in mice, Clevers and coworkers identified stem cells in the small intestine and colon using the marker gene *Lgr5* [28] and proposed them as the cells-of-origin of intestinal cancer [23]. At the same time, Sangiorgi and Capecchi’s study found another intestinal stem cell marker in vivo, Bmi1 [24]. Importantly, Bmi-1 and Lgr5 markers define two types of SCs, quiescent and rapidly cycling SCs, respectively [23,24], and may identify CCSCs. Vermeulen et al. showed that spheroid cultures from primary CRC have a tumor-initiating capacity and that a cell subpopulation expresses CD24, CD29, CD44 and CD166 markers, suggested as CCSC markers [25]. The study by Pang et al. identifies a subpopulation of CD26^+^ cells capable of developing distant metastases when injected into the mouse cecal wall and associated with increased invasiveness and chemoresistance, whereas CD26^−^ cells cannot [26]. Interestingly, the presence of CD26^+^ cells in the primary tumor of patients without distant metastases at that time may predict future distant metastases, highlighting a critical role of CSCs in the progression of metastatic cancer and important clinical implications [26]. The transmembrane glycoprotein CD44 has several splicing variants, including CD44v6, which appears to negatively impact the prognosis of CRC patients [29,30]. Todaro et al. demonstrated that all identified CCSCs express the CD44v6 marker, which supports their migration and promotes metastasis [27]. Each of these markers has its own function and role in the prognosis of CRC, as shown in Table 2.

All these markers can be expressed by CCSCs, but they do not all have the same capacity. Some, such as CD133, Lgr5, Bmi-1, CD26 and CD44v6 alone identify CCSCs, while the other presented markers allow the identification of CCSCs only in combination with one or more of the aforementioned markers. In conclusion, these markers play a key role in the identification of CCSCs and can be used alone or in combination to sort CCSCs by magnetic-activated cell sorting (MACS) or fluorescence-activated cell sorting (FACS) techniques.

**Figure 1:**
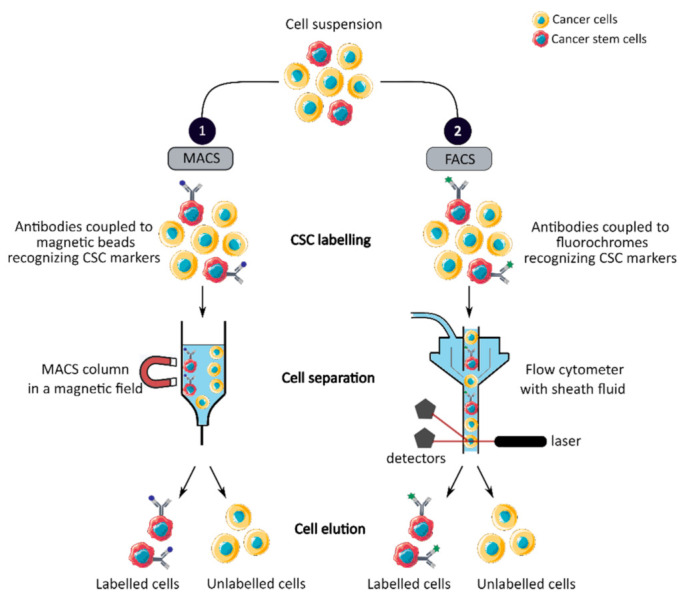
Phenotypic sorting of CSCs through the expression of CSC markers recognized by antibodies coupled to either magnetic beads, MACS (1), or fluorochromes, FACS (2). Once the antibodies are added, the cell suspension is passed through either a MACS column in a magnetic field that retains the antibody-labeled cells (1) or through a flow cytometer that distinguishes and isolates labeled cells from unlabeled cells (2). CSC: cancer stem cell; MACS: magnetic-activated cell sorting; FACS: fluorescence-activated cell sorting.

MACS is a magnetic-based cell isolation technique, using a positive selection strategy, presented in Figure 1 panel 1 [44]. Magnetic beads are conjugated to highly specific monoclonal antibodies that recognize CCSC marker on the surface of cells of interest. Then, the heterogeneous suspension of cells is passed through a separation column, in a magnetic field, to retain the cells labeled with magnetic beads and antibodies [45]. By switching off the magnetic field, target cells will be eluted. MACS is a fast and easy method of cell separation, especially for the isolation of CCSCs that represent a small cell population in the tumor mass. However, MACS is only a mono-parameter separation method that requires cell labelling and is unable to separate cells based on the variable expression of markers [44,45].

FACS uses fluorescently labeled antibodies that target the cell surface or intracellular markers to isolate CCSCs [44]. Antibodies are conjugated to fluorochromes and recognize the marker of interest within a cell suspension, as shown in Figure 1 panel 2 [44]. The cell suspension is then hydrodynamically focused into a stream of individual cells by the flow cytometer and passed through a laser which provides information on the size, granularity and fluorescent properties of single cells [18]. Fluorochromes with different emission wavelengths can be used simultaneously to allow multiparameter separations [44]. Both technologies allow the sorting of CCSCs with high purity but require the availability of antibodies and cell labeling, which can modify their properties and induce cell differentiation [16,44,46]. In addition, phenotypic characterization is insufficient to define a CCSC because these markers are also expressed by normal SCs.

Therefore, in order to confirm the detection and isolation of CCSCs, their functional capabilities need to be evaluated by in vitro and in vivo assays [18].

#### 2.2.2. CCSC Isolation Based on Functional Features

**Figure 2:**
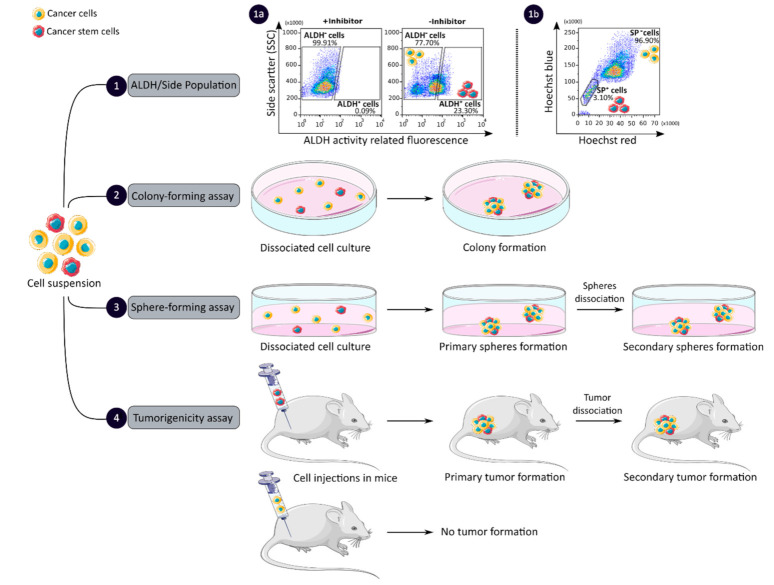
Functional sorting of CSCs due to their specific properties such as enhanced detoxification (1), ALDH (1a) and SP (1b), in vitro self-renewal and differentiation capacity, colony- (2) and sphere-forming (3) assays, and the ability to form tumors in vivo, tumorigenicity assay (4). CSC: cancer stem cell; ALDH: aldehyde dehydrogenase; SP: side population.

CCSCs have many intrinsic properties that can be used to identify them, such as their capacity for self-renewal, multi-lineage differentiation, detoxification due to aldehyde dehydrogenase 1 (ALDH1) activity and dye exclusion ability, colony/sphere formation and tumorigenicity, which are illustrated in Figure 2. These functional characteristics have been used to develop effective methods for isolating CCSCs. The ALDH activity assay is based on the use of a fluorescent and non-toxic ALDH substrate that freely diffuses into intact and viable cells [47]. Then, in the presence of the detoxifying enzyme ALDH, the substrate is converted into a negatively charged fluorescent product that is retained inside the cells. Thus, cells with high ALDH activity become brightly fluorescent and can be measured by flow cytometry as presented in Figure 2 panel 1a [47,48]. CCSCs increase their ALDH1 activity to resist to chemotherapeutic agents and prevent apoptosis by maintaining low levels of reactive oxygen species [47]. The advantage of the ALDH assay is high stability compared to the use of surface markers, but its specificity is low due to its expression in both normal SCs and CSCs [48].

The side population (SP) assay relies on the differential ability of the cells to efflux dye via ATP-binding cassette (ABC) transporters [49]. Hoechst33342 is a fluorescent dye that binds all nucleic acids and has the particularity of passing through the plasma membrane of living cells. When excited by UV lights, Hoechst dye emits a fluorescence that can be detected by a flow cytometer [49]. SP cells are capable of actively removing the dye from the cell and have a unique low Hoechst fluorescence emission, as shown in Figure 2 panel 1b. CCSCs highly express efflux transporters, such as multidrug resistance protein 1 (ABCB1), multidrug resistance-associated proteins (ABCC1) and breast cancer resistance protein (ABCG2), to protect themselves against cytotoxic substances and therefore look like SP cells [18]. The SP assay is an easy and reliable method that does not require cell labeling, but due to its low purity and specificity, the SP assay is often combined with cell labeling to significantly increase the purity of sorted CSCCs [18,49].

Colony and sphere formation assays evaluate in vitro the self-renewal and differentiation capacities of individual cells in two (2D) and three (3D) dimensions, respectively, which are shown in Figure 2, panels 2 and 3 [50,51]. Both assays are based on non-adherent cultures using either a soft agar layer (2D) or low adherent plates (3D) [52,53]. In the soft agar method illustrated in Figure 2 panel 2, the suspension of individual cells is mixed with the soft agar which may, after several weeks of incubation, give colonies that can be stained with crystal violet to determine their number and size [50,52]. In comparison, in the 3D culture shown in Figure 2 panel 3, the individual cells in suspension are grown at very low cell density and in serum-free medium (DMEM/F12 medium) supplemented with growth factors (human recombinant basic fibroblast growth factor and human recombinant epidermal growth factor), N2 supplement, glucose, insulin and optionally antibiotics such as penicillin/streptomycin for several weeks to obtain spheroids [51,54]. The produced spheroids mimic various characteristics of solid tumors, such as growth kinetics, gene expression pattern and cellular organization with the outer layer containing highly proliferative cells, the middle layer with senescent or quiescent cells and the inner layer comprising necrotic cells due to a lack of oxygen and nutrients [53]. CCSCs can be identified in both techniques as they have the ability to form larger and more numerous colonies and are capable of giving rise to a tumor sphere (colonosphere) resembling the primary sphere when passed in series, due to their ability to grow and divide independently of their environment which normal cells are unable to do because of anoikis [18,52,55]. Thus, in vitro, 3D models appear to be a relevant preclinical model for testing new drugs, evaluating potential combinations and understanding drug resistance, by mimicking CSC-containing tumors in vitro, before testing them in vivo [18,53,55]. However, these models require well-established protocols and appropriate cell dilution to certify that each colony/sphere is derived from a single cell [18].

The tumorigenicity assay is considered the gold standard method for studying the CSC properties of human tumors in vivo [18,56]. This approach allows to determine the tumor-initiating ability of cancer cells in immunodeficient mice and their capacity for self-renewal in vivo after the dissociation of primary tumors and transplantation in secondary recipient mice, as illustrated in Figure 2 panel 4 [57]. In vivo limiting dilution is the best method for identifying the lowest concentration of cells capable of forming a tumor and determining the frequency of CSCs [18,58]. Importantly, only CSCs have the ability to generate a xenograft that is histologically similar to the parental tumor from which it originated, to be serially transplanted in a xenograft assay due to their long-term self-renewal capacity, and to generate daughter cells [56,58]. However, the use of mouse models requires ethical consideration and complicated laboratory equipment. In addition, the results of xenograft experiments are highly dependent on the number of cells, the implantation site and the incubation period, which leads to certain limitations [18]. Nevertheless, mouse models remain unique models for studying the biology of CSCs in vivo [57,58].

#### 2.2.3. CCSC Isolation Based on Biophysical Features

**Figure 3:**
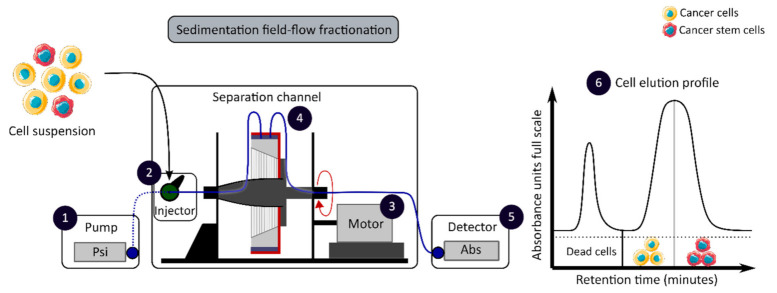
Biophysical sorting of CSCs according to their size, density, shape and rigidity using the SdFFF technique, which does not require cell labelling or fixation. The SdFFF is composed of a pump (1) to transport the mobile phase (PBS) and the cells, an injector (2) to introduce the cell suspension, a motor (3) to rotate the separation channel (4) and a detector (5) coupled to a computer to obtain the elution profile of the cell suspension (6). Psi is a common unit of pressure. CSC: cancer stem cell; SdFFF: sedimentation field-flow fractionation; PBS: phosphate-buffered saline; Abs: absorbance.

The development of enrichment and isolation methods for CCSCs without cell labeling offers new perspectives, such as sorting techniques based on biophysical characteristics. The sedimentation field-flow fractionation (SdFFF) is a gentle, non-invasive and label-free method that prevents interference for further cell use and the allows separation of cells according to their size, density, shape and rigidity [16,59]. Cell separation by SdFFF depends on the differential elution of cell subpopulations submitted both to the action of a parabolic profile generated by the mobile phase in the channel and to a multigravitational external field generated by the rotation of the channel, as presented in Figure 3 [16,59]. In the past decade, SdFFF cell sorting has been adapted and applied in many fields such as neurology, oncology and stem cells [16,60,61,62]. The study by Mélin et al. describes a strategy, based on SdFFF elution, to obtain activated and quiescent CSC subpopulations from eight different human CRC cell lines [16]. The combination of cell sorting by SdFFF with the grafting of these CSC-enriched fractions into chick embryo chorioallantoic membrane (CAM) model demonstrates the potential of SdFFF to produce innovative matrices for the study of carcinogenesis and the analysis of treatment sensitivity [16,63]. The advantages of this isolation method are the use of biophysical characteristics for cell sorting without cell labeling; however, this technique requires a large number of cells and is time consuming [46].

#### 2.2.4. CCSC Isolation Methods: Discussion

Taken together, this chapter provides an overview of the techniques commonly used to identify and sort CCSCs, which are summarized in Table 3. The use of cell surface markers remains the most widely used in cancer research, however, it remains controversial due to the lack of a universal marker for CCSCs. Moreover, nowadays, none of the CSC isolation techniques are capable of 100% enrichment of CCSCs due to the shared properties between normal SCs, non-CCSCs and CCSCs [14,17]. As an example, Shmelkov and colleagues have shown that CD133 expression in the colon is not limited to SCs but is also expressed on differentiated tumors cells [64]. In addition, the authors found that both CD133^+^ and CD133^−^ isolated from metastatic colon tumors are capable of initiating tumors in a serial xenotransplantation model [64]. A few years later, the study by Kemper et al. demonstrated that CD133 is expressed on the cell surface of CSCs and differentiated tumor cells but is differentially glycosylated [21]. Similarly, using the ALDH activity assay, Huang et al. found that ALDH1 is a marker of both normal and malignant human colonic SCs [48]. Consequently, cell surface markers and ALDH activity cannot be used alone to sort and define CSCs. Thus, the SdFFF technique offers new perspectives for CSC sorting that does not require cell labeling or fixation and thereby allows the combination of this technique with other CSC characterization methods. Therefore, the combined use of CCSC isolation methods can provide a more powerful and efficient tool for identifying and sorting CCSCs. The advantages and weaknesses of each method must be known in order to select the best method based on the experimental question, as shown in Table 3.

## 3. Clinical Relevance of Colorectal Cancer Stem Cells

Therapeutic advances made in recent decades now enable most cancer patients to achieve major clinical responses [6]. However, although therapeutic approaches are increasing, none of these treatment modalities is curative in most cases of advanced CRC [65]. Furthermore, despite initially successful treatment reflecting the therapeutic effect on the cells that form the tumor bulk, tumor recurrence is almost inevitable due to the development of drug resistance attributed to CCSCs [8].

### 3.1. Clinical Management of Colorectal Cancer

Treatment options and recommendations depend on several factors, including the patient’s overall health, possible side effects, the type and stage of the tumor, and its mutational and MMR status [1,5]. Therapeutic approaches for the treatment of CRC include surgical resection, local therapies for metastatic disease, systemic therapy comprising chemotherapy, targeted therapy and immunotherapy as presented in Table 4, and palliative chemotherapy [6]. To ensure the optimal survival and quality of life for patients, personalized therapy is crucial to enable cancer patients to maximize the benefits while minimizing the harms [5].

Surgical resection is the mainstay of curative intent treatment for localized and advanced CRCs but needs to be complete to be considered curative when there is regional invasion or histological factors with a poor prognosis [66,67]. Surgery can be associated with neoadjuvant therapy in order to shrink tumor mass and facilitate medical operation and/or with adjuvant therapy to limit cancer recurrence [1]. Importantly, neoadjuvant chemotherapy, possibly coupled with radiotherapy, is mainly indicated for rectal cancers [68]. Treatment regimens for patients with localized CRC generally include chemotherapy such as 5-fluorouracil (5-FU) or capecitabine, oxaliplatin and irinotecan, alone or in combination [69,70,71,72,73]. Leucovorin is commonly administered with 5-FU to enhance its anti-tumor effect [74]. Despite many advances in CRC treatment, approximately 20% of new CRC cases are already metastatic [75]. The most common sites of metastatic colorectal cancer (mCRC) are the liver, lungs and peritoneum. Unfortunately, up to 50% of patients with early-stage disease at diagnosis will eventually develop metastatic disease, and 80–90% of them have unresectable metastatic disease because of the size, location, and/or extent of disease [76,77].

Local therapies are approved for mCRC with inoperable lesions. The choice of local therapies depends on the location and the extent of the metastases [78]. For patients with unresectable liver or lung metastases, radiofrequency ablation is recommended for the treatment of small and medium-sized lesions, but for larger lesions and those near vascular structures, microwave ablation or stereotactic body radiation therapy may be good alternatives [1,6]. Liver metastases can also be treated by administering a higher dose of chemotherapy directly into the hepatic artery compared to systemic therapy (hepatic arterial infusion) or by combining drug/radiation administration with blood vessel obstruction (chemo/radio-embolization) [79]. For patients with peritoneal metastases, cytoreductive surgery and hyperthermic intraperitoneal chemotherapy are recommended [6]. Local therapies can be administered with curative or palliative intent and are the most often used in combination with systemic therapy [6,79].

Systemic therapy for CRC aims to downsize the primary tumor or metastases in order to convert them to a resectable status and increase progression-free survival [6]. Patients with advanced CRC usually receive several lines of therapy, most often including a combination of chemotherapy with targeted therapy or immunotherapy, depending on tumor mutational and MMR status [5]. Targeted therapies are recommended for patients with *KRAS/NRAS/BRAF* mutated or wild-type tumors, *HER2*-amplified tumors and *NTRK* gene fusion-positive tumors, while immunotherapy is only offered for tumors with high microsatellite instability (MSI), as shown in Table 4. Thus, both statuses must be determined prior to the start of therapy [80]. Unfortunately, for advanced CRC patients whose overall health has deteriorated despite treatment, palliative treatments and the best supportive care are the only remaining options [5]. Therefore, the clinical management of patients with mCRC represents a unique challenge to balance benefits and harms, including the identification of strategies that improve disease response, limit treatment-associated toxicities, and improve the overall quality of life [81].

### 3.2. Mechanisms of Drug Resistance Associated with Colorectal Cancer Stem Cells

**Figure 4:**
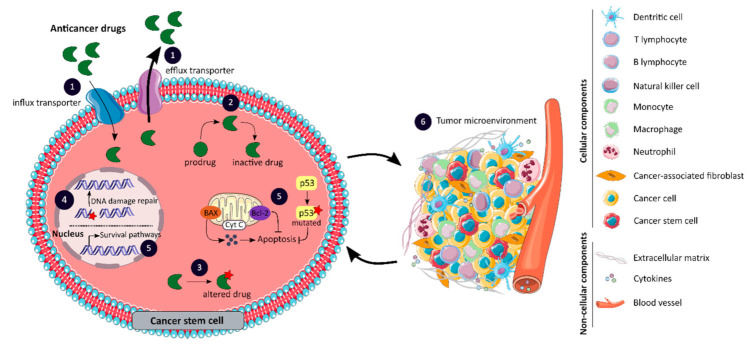
Major mechanisms of anticancer drug resistance attributed to CSCs such as changes in drug transport (1); impaired drug metabolism (2); alterations in drug targets (3); enhanced DNA damage repair (4); impaired balance between apoptosis and survival pathways (5); and the role of the tumor microenvironment comprising cellular and non-cellular components (6). CSC: cancer stem cell; DNA: deoxyribonucleic acid.

The effectiveness of current anticancer therapies is limited by the resistance of tumors to chemotherapy and targeted molecular therapies [99]. Resistance to anticancer drugs may be intrinsic, meaning that it occurs prior to treatment and involves pre-existing resistance factors in the mass of tumor cells, or it may be acquired during the treatment of tumors that were initially sensitive due to the induction of various adaptive responses [99]. Furthermore, due to the high degree of tumor heterogeneity, drug resistance may also result from the therapy-induced selection of a drug-resistant tumor subpopulation, such as CCSCs [99]. A wide range of molecular mechanisms are involved in drug resistance, as illustrated in Figure 4, and will be detailed in this chapter [74].

#### 3.2.1. Changes in Drug Transport

The anticancer activity of a drug can be limited by poor drug influx or excessive efflux, which alters the amount of drug reaching the tumor, as shown in Figure 4 panel 1 [99]. Several transporter proteins, belonging to the superfamilies ABC and solute carrier (SLC), have been linked to anticancer drug resistance by interfering with drug transport [74]. The ABC transporters ABCB1, ABCC1 and ABCG2 play a pivotal role in the efflux of anticancer drugs [100,101]. In colon cancer, ABCB1 may be overexpressed, leading to reduced cellular accumulation of chemotherapy and therefore therapeutic failure, or may be induced by chemotherapy resulting in the acquired development of multidrug resistance [99]. The impact of SLCs on cancer therapy has been less documented, however, some members of the SLC superfamily are also involved in the transport of anticancer drugs [100]. Changes in the expression of SLC transporters, such as the organic cation transporter OCT2 and the organic zwitterion/cation transporters OCTN1, may affect the ability of tumor cells to uptake anticancer drugs and lead to the development of chemoresistance [100]. The Zhang et al. study shows that the overexpression of human OCT2 transporters increases oxaliplatin accumulation and cytotoxicity in colon cancer cell lines [102]. Taken together, efflux and influx transporters may confer resistance to anticancer agents, and the intrinsic drug resistance of CCSCs may be explained by the higher expression of these transporters [99,100,102].

#### 3.2.2. Impaired Drug Metabolism

The efficacy of anticancer drugs may also be affected by changes in their metabolism, such as the production of an inactive metabolite, as highlighted in Figure 4 panel 2 [99]. The inactivation of anticancer drugs may be associated with the overexpression of drug-metabolizing enzymes, such as cytochrome P450-related enzymes (CYP), UDP-glucuronosyltransferase (UGT) and glutathione S-transferase (GST) [74]. CYP enzymes play a crucial role in the metabolism of many therapeutic drugs, including SN-38, the active metabolite of irinotecan. Indeed, SN-38 can be inactivated by CYP3A4- and CYP3A5-dependent oxidations that form inactive metabolites [103]. The study by Buck et al. shows a significant correlation between CYP3A5 expression and tumor response to irinotecan therapy in CRC [103]. In addition, increased CYP expression in CSCs appears to be associated with chemoresistance [104]. SN-38 is predominantly eliminated by glucuronidation which is mainly mediated by the polypeptide A1 of the UGT1 family, encoded by the *UGT1A1* gene [105]. However, inter-individual variations in UGT1A1 activity exist and are related to the presence of genetic polymorphisms. For example, patients with UGT1A1*28/*28 genotype have a higher risk of developing irinotecan-induced hematological toxicity and require a reduction in irinotecan dose which may impact its anti-cancer effect [105]. The GSTP1 subclass of the GST superfamily is overexpressed in patients with colon cancer and is an important mediator of intrinsic and acquired platinum resistance [106]. Stoehlmacher et al. demonstrated that GSTP1 Ile^105^Val polymorphism is associated with increased survival in patients with advanced CRC receiving 5-FU/oxaliplatin chemotherapy [106]. Thus, the enhanced ability of tumor cells, particularly CCSCs, to inactivate anti-cancer drugs is mainly due to the overexpression of drug-metabolizing enzymes or polymorphisms [74].

#### 3.2.3. Alterations in Drug Targets

One of the most common mechanisms of resistance to targeted therapy is mediated by alterations in the target protein as suggested in Figure 4 panel 3 [107]. Somatic mutations have been identified in the *KRAS* gene as a biomarker of intrinsic resistance to EGFR-targeting agents in patients with CRC [108]. The Misale et al. study reports for the first time that a substantial fraction of CRC patients who exhibit an initial response to anti-EGFR therapies have, at the time of disease progression, tumors with focal amplification or somatic mutations in *KRAS* which were not detectable prior to therapy initiation [108]. Thus, drug resistance resulting from *KRAS* alterations can be attributed not only to the selection of pre-existing *KRAS* mutant and amplified clones, but also to new mutations resulting from ongoing mutagenesis [108]. The acquisition of mutations in target proteins also contributes to chemotherapy drug resistance. Irinotecan exerts its cytotoxic activity by inhibiting topoisomerase 1 (TOP1). However, increased TOP1 gene copy number at 20q11.2-q13.1 or mutations in the gene that result in reduced affinity for its active metabolite may be involved in increased drug resistance in CCR [74]. Therefore, the alteration of drug targets primarily due to the acquisition of mutations may result in resistance to targeted therapy and chemotherapy.

#### 3.2.4. Enhanced DNA Damage Repair

Drug resistance can also be explained by an enhanced ability of tumor cells, especially CCSCs, to repair drug-induced DNA damage, as presented in Figure 4 panel 4. The repair of DNA adducts induced by platinum-based chemotherapy, such as oxaliplatin, is primarily mediated by the nucleotide excision repair (NER) pathway [74]. The upregulation of excision repair cross-complementing 1 (ERCC1), a key protein of the NER pathway, has been associated with oxaliplatin resistance in CRC [74]. In addition, the level of intra-tumoral ERCC1 mRNA expression is a predictive marker of survival in mCRC patients receiving combination chemotherapy with 5-FU and oxaliplatin [109]. Mismatched or wrongly matched nucleotides are corrected by the MMR system, which plays a crucial role in maintaining genome integrity [74]. DNA repair deficiency can be caused by mutations in *MMR* genes, such as *MLH1* and *MSH2,* and can lead to the MSI phenotype [99]. The study by Valeri et al. shows that the microRNA-21 (miR-21) downregulates hMSH2, and miR-21 overexpression reduces the therapeutic efficacy of 5-FU in a CRC xenograft model, suggesting that the downregulation of MSH2 with miR-21 overexpression may be an important indicator of therapeutic efficacy in CRC [110]. Consequently, the defects or upregulation of DNA repair pathways can serve as biomarkers of therapeutic response, and therapeutic effects can be enhanced by combining the inhibition of a DNA damage response pathway with DNA-damaging agents to eradicate CCSCs [111].

#### 3.2.5. Impaired Balance between Apoptosis and Survival Pathways

Resistance to cell death is one of the hallmarks of human cancers that contribute to tumor progression and drug resistance [101]. Cell death by apoptosis is a physiological program controlled by a tightly regulated balance between pro-apoptotic, anti-apoptotic and pro-survival mechanisms [112]. However, this balance is frequently altered in cancer cells and particularly in CCSCs, as shown in Figure 4 panel 5. The tumor suppressor p53, encoded by the *TP53* gene, is essential for the induction of apoptosis in response to chemotherapy [74]. Nevertheless, p53 is found mutated in approximately 85% of CRC cases, and *TP53*-mutated colon cancer cells tend to be more resistant to many anticancer drugs, including 5-FU and oxaliplatin, compared to *TP53* wild-type cells [74,101]. The BCL-2 family, which contains pro- and anti-apoptotic members, plays a crucial role in the regulation of apoptosis. The loss of expression and/or activity of the pro-apoptotic factor BAX can be explained by frameshift mutations in the *BAX* gene and may result in chemoresistance [74]. The study by Nehls et al. suggests a major prognostic impact of BAX, whose protein expression appears to be important for the clinical outcome of 5-FU-based adjuvant chemotherapy in stage III colon cancer [113]. The balance between apoptosis and survival may also be altered by aberrantly overexpressed or overactivated anti-apoptotic factors, such as Bcl-2, Bcl-XL, the inhibitor of apoptosis proteins and the caspase 8 inhibitor FLIP [74,99]. Importantly, alterations in the genes encoding these anti-apoptotic factors have been linked to resistance to chemotherapy and targeted therapy [99]. Finally, the overactivation of several pro-survival signaling pathways, including Notch, Hedgehog, Wnt, Bone morphogenetic proteins, Janus kinase/signal transducers and activators of transcription (JAK/STAT) and nuclear factor-κB pathways, may also be associated with drug resistance [112]. Taken together, the altered balance between apoptosis and survival in cancer cells, and especially in CCSCs, prevents apoptosis even when DNA repair fails, which is another mechanism of resistance to therapy [112].

#### 3.2.6. Role of the Tumor Microenvironment

In recent years, the TME has emerged as a key driver of tumor progression and drug resistance, challenging the development of new therapies in clinical oncology. TME contains both cellular components with cancerous and non-cancerous cells such as stromal myofibroblasts, endothelial cells, immune cells and cancer-associated fibroblasts (CAFs), and non-cellular components including extracellular matrix (ECM), cytokines, growth factors and extracellular vesicles, as illustrated in Figure 4 panel 6 [114]. In the tumor stroma, CAFs secrete the cytokines CXCL1 and CXCL2 as well as the interleukin-6, which promote angiogenesis and tumor progression [46,114]. Vermeulen et al. showed that myofibroblast-secreted factors, in particular hepatocyte growth factor (HGF), enhance Wnt signaling activity in colon cancer cells and can restore the CSC phenotype in more differentiated tumor cells, both in vitro and in vivo [115]. Furthermore, CSCs reside in anatomically specialized regions of the TME, known as the CSC niche, which retain their properties and protect them from anticancer drugs, contributing to their enhanced resistance to treatment [46,114,116]. Importantly, CSCs can also be maintained in a quiescent state with minimum energy consumption and a low proliferation rate to resist therapies [114]. In response to environmental signals such as hypoxia, the niche adapts to ensure optimal conditions for CSC proliferation and differentiation [46]. CSCs may contribute to vessel recruitment during tumorigenesis by secreting angiogenic factors, such as vascular endothelial growth factor (VEGF) and CXCL12, in order to accelerate angiogenesis in endothelial cells, which in turn secrete factors such as nitric oxide and osteopontin to maintain the stemness of CSCs [15]. Hypoxia is a key factor in cancer progression that regulates cell survival, angiogenesis, invasion and metastasis, via hypoxia-inducible factor (HIF) [14,116]. In addition, hypoxia can induce the epithelial-to-mesenchymal transition (EMT) that leads to the dissemination and invasion of tumor cells due to the loss of cell adhesion properties and the acquisition of a mesenchymal phenotype with motility and invasiveness [8,116]. The expression of SNAI1 protein, the main inducer of EMT, has been detected at the tumor–stromal interface in colon cancer [116] and elevated endogenous levels of SNAI1 in cancer cells have been shown to increase tumor initiation capacity and metastatic potential in mouse and human models [8].

#### 3.2.7. Mechanisms of Drug Resistance Associated with CCSCs: Discussion

Several publications point out that one of the main technical issues in the CSC field is the plasticity of CCSCs and tumor cells, which may be involved in drug resistance [117,118,119,120]. Using the CRISPR-Cas9 technology, Shimokawa et al. demonstrated that the selective ablation of LGR5^+^ CCSCs in human CRC organoids leads to tumor regression in xenografts produced by these organoids [120]. However, after several weeks, tumor regrowth is observed and associated with differentiated tumor cells that dynamically replenish the pool of LGR5^+^ CCSCs, indicative of cellular plasticity [120]. Another study confirmed these results using CRC organoids that express the diphtheria toxin receptor under the control of the LGR5 locus to selectively ablate LGR5^+^ CCSCs [117]. Importantly, the removal of CCSCs limits primary tumor growth but does not prevent the regrowth of the tumor at the primary tumor site upon discontinuation of treatment due to proliferative LGR5^−^ cells, whereas it does in metastatic lesions [117]. Thus, the authors demonstrated a protective role of selective CSC depletion in primary tumors on the appearance of distant metastases, suggesting an interesting therapeutic perspective for the management of metastatic diseases [117]. The process of cellular plasticity is crucial for the repopulation of impaired SC niches and tissue homeostasis, but its role in the formation of metastases is poorly studied [118]. Using a CRC mouse model and human tumor xenografts, Fumagalli et al. investigated the role of cellular plasticity in metastasis [118]. Surprisingly, the authors show that the majority of disseminated CRC cells in the circulation are LGR5^−^ cancer cells and are capable of forming distant metastases, in which LGR5^+^ CSCs subsequently emerge and contribute to long-term metastatic growth [118]. Importantly, microenvironmental factors may enhance cellular plasticity [118]. Thus, cellular plasticity complicates the development of new therapeutic strategies and the eradication of CCSCs does not appear to be sufficient to completely cure cancer due to the impact of the microenvironment [8]. The heterogeneous and dynamic nature of SCCCs constitutes another obstacle to their targeting. Using a marker-free and quantitative analysis of colon cancer growth dynamics, Lenos et al. showed that cells with CSC functionality are not necessarily the same cells as those expressing CSC markers [121]. Interestingly, the authors demonstrated that all tumor cells have the capacity to fuel tumor growth when placed in an appropriate environment, preferentially at the edge of the tumor close to the CAFs [121,122]. Thus, from the authors’ point of view, the stem cell function in established cancers is not intrinsically but entirely spatiotemporally orchestrated, suggesting a major role of the microenvironment [121]. Consequently, cellular plasticity and the microenvironment appear to allow tumors to easily adapt to the loss of key compartments, thereby compromising therapeutic efficacy [122]. Therefore, TME plays a crucial role in primary tumor growth and metastasis formation by protecting CSCs from therapeutic agents and appears to be an important target along with the other resistance mechanisms discussed in this chapter for the development of new therapies [116].

## 4. Clinical Trials on Colorectal Cancer Stem Cells

Clinical trials targeting CCSCs are rare. The complexity relies on the identification of molecular targets required to maintain cancer stemness in CSCs, but not or less by normal tissue SCs, to selectively target CSCs [123]. All clinical trials from the National Institute of Health are listed on the ClinicalTrials.gov website [124]. We narrowed our search by focusing on the terms "colorectal cancer" and "cancer stem cells", resulting in the identification of eight intervention studies as of September 30, 2020. Among them, we excluded all clinical trials whose status was withdrawn (N = 1) and terminated (N = 2) and focused on the remaining clinical trials (N = 5). Subsequently, from these five clinical trials, we selected and reviewed the clinical trials on pharmacological agents under investigation (N = 3), as presented in Table 5.

Napabucasin (BBI608) is the first-in-class cancer stemness inhibitor that targets the STAT3 pathway [123,125]. In a preclinical study, Li et al. showed that BBI608 inhibits the expression of stemness genes and the self-renewal of CSCs and succeeds in depleting CSCs whereas standard chemotherapy leads to the enrichment of these cells [123]. In addition, the authors demonstrated the ability of BBI608 to block both cancer relapse and metastasis in vivo, using a mouse CRC model [123]. These preclinical results provide an interesting approach for the development of new anticancer therapies targeting cancer stemness [123,125]. Several clinical trials were designed prior to the ongoing phase III clinical trial, shown in Table 5. Firstly, a phase I dose-escalation study was conducted in adult patients with advanced malignancies who had failed standard therapies in order to investigate the safety and anti-tumor activity of BBI608 as monotherapy (NCT01775423) [126,127]. BBI608 showed encouraging signs of clinical activity with only mild adverse events observed and an unreached maximum tolerated dose (MTD), suggesting an excellent safety profile of BBI608 at 500 mg twice daily [126,127]. Subsequently, two additional phase Ib/II clinical trials were conducted to determine the safety and anti-tumor activity of BBI608 in combination with panitumumab in *KRAS* wild-type patients with mCRC (NCT01776307) or with FOLFIRI (5-FU/leucovorin/irinotecan) +/− bevacizumab in mCRC (NCT02024607). Both clinical trials showed a high disease control rate (DCR) including patients with partial response, stable disease or tumor regression, which confirms the safety of these combinations with encouraging anti-tumor activity [128,129,130]. Thereafter, a phase III study was designed to evaluate the efficacy and safety of BBI608 versus placebo with the best supportive care in patients with advanced CRC who had failed all available standard therapy (NCT01830621) [131]. In this trial, BBI608 did not improve overall survival (OS) or progression-free survival (PFS) in unselected patients with advanced CRC but did improve OS in pSTAT3-positive patients compared to the placebo group, suggesting that STAT3 may be an important target for CRC treatment [131,132]. Finally, the ongoing phase III clinical trial aims to assess the efficacy of BBI608 plus FOLFIRI versus FOLFIRI alone in previously treated mCRC patients (N = 1250) (NCT02753127) [133]. Patients are randomized 1:1 in each group and stratified by time to progression to first-line therapy, *RAS* mutation status and primary tumor location [133]. The endpoints of this clinical trial are OS, PFS, DCR and objective response rate in both the general population and p-STAT3-positive subpopulation [133].

Demcizumab (OMP-21M18) is a humanized anti-DLL4 (delta-like ligand 4) antibody that inhibits the Notch pathway and CSC activity through the inhibition of tumor growth and reduction in tumor-initiating cell frequency [134,135,136]. The study by Ridgway et al. shows that treatment with a DLL4-selective antibody disrupts tumor angiogenesis and inhibits tumor growth in several mouse tumor models [137], these results were confirmed by Hoey et al. using xenograft models of colon tumors [136]. In addition, treatment with anti-human DLL4, alone or in combination with irinotecan, delays tumor recurrence and reduces the frequency of CSCs, as demonstrated by the limiting dilution assay and in vivo tumorigenesis studies [136]. As a result of these preclinical results, several clinical trials were conducted with OMP-21M18. A phase I dose-escalation study was designed to determine the safety, MTD and pharmacokinetics of OMP-21M18 in patients with a previously treated solid tumor for which there is no remaining standard curative therapy (NCT00744562) [138]. In this trial, no more than one dose-limiting toxicity (DLT) was observed at each dose, corresponding to the appearance of side effects severe enough to prevent an increase in the dose of the drug, and the MTD was not reached [138]. OMP-21M18 was generally well tolerated by patients at doses below 5 mg per week and showed anti-tumor activity highlighted by the stabilization of the disease and decrease in tumor size. However, the prolonged administration of this drug was associated with an increased risk of congestive heart failure [138]. Subsequently, a phase Ib study failed to demonstrate enhanced anti-tumor activity of OMP-21M18 in combination with the anti-PD-1 pembrolizumab in patients with advanced or metastatic solid tumors, despite the fact that the combination therapy was well tolerated (NCT02722954) [139]. Finally, a phase I study was conducted to determine the safety and optimal dose of OMP-21M18 in combination with FOLFIRI in patients with mCRC (N = 32) (NCT01189942). Safety was scheduled to be assessed in each patient group after 56 days of treatment and disease status every 8 weeks. The endpoints of this clinical trial were to determine the MTD of OMP-21M18 plus FOLFIRI and to evaluate the safety, pharmacokinetics and preliminary efficacy of this combination. Unfortunately, to date, no results from this clinical trial have been found, although the actual completion date of the study indicated on ClinicalTrials.gov is February 2011.

Mithramycin A (Mit-A) is an antineoplastic antibiotic agent and a potent inhibitor of specificity protein 1 (SP1) [140]. In various human malignancies, SP1 is overexpressed and contributes to the malignant phenotype by regulating genes involved in proliferation, invasion, metastasis, stemness and chemoresistance [141,142]. The study by Zhao et al. demonstrates that the inhibition of SP1 by Mit-A suppresses the growth of colon CSCs and attenuates their characteristics by significantly reducing the percentage of CD44^+^/CD166^+^ cells in vitro and in vivo [142]. Another study shows that Mit-A inhibits tumor growth and significantly induces cell death and the PARP cleavage of CSC and non-CSC cells [140]. Thus, these preclinical results highlighted Mit-A as a potentially promising drug candidate for the treatment of CRC [140]. Several clinical trials have been conducted to investigate the safety and efficacy of Mit-A in chest cancers, solid tumors and Ewing sarcoma (NCT01624090 and NCT01610570) [143]. Despite the promising preclinical activity of Mit-A in various advanced malignancies, several patients developed severe hepatotoxicity due to the altered expression of hepatocellular bile transporters resulting in the early termination of the clinical trial [144]. The objective of the ongoing phase I/II clinical trial NCT02859415 was to determine the safest dose of Mit-A in patients with chest cancers, including CRC patients, by specifically selecting patients without these alterations. The endpoints of this clinical trial are to evaluate the DLT, MTD, and pharmacokinetics of Mit-A in patients with primary thoracic malignancies or carcinomas, sarcomas or germ cell neoplasms with pleuropulmonary metastases and to determine their overall response rates.

Clinical Trials on CCSCs: Discussion

Thus, the development of therapeutic agents specifically targeting CCSCs is complex, as outlined in this chapter. Unfortunately, despite encouraging preclinical results, the majority of ongoing clinical trials fail to demonstrate relevant results in phase I/II development, which examines the safety of the drug, and does not allow them to proceed to the next phase. In our search of ClinicalTrials.gov, we found only three clinical trials focusing on CSCs and recruiting patients with CRC, underscoring the rarity and complexity of clinical trial design [124]. Among these trials, no study results were found for one of the drugs tested, demcizumab, although the actual completion date of the study has passed [139]. In addition, of the three drugs in clinical trials, two drugs showed drug-related toxicities in the current or previous study. The prolonged administration of demcizumab was associated with an increased risk of congestive heart failure [138] and some patients treated with Mit-A developed severe hepatotoxicity [144]. However, one of these three drugs, napabucasin, has shown interesting results in previous clinical trials and is currently in a phase III study [128,129,130]. In conclusion, the development of clinical trials can encounter many problems related either to drugs, to patients with unexpected side effects or toxicities, or to the design of the study.

## 5. Future Perspectives

The main challenge in preclinical studies is to obtain relevant results that translate into meaningful clinical activity in patients with CRC [134]. Unfortunately, despite interesting preclinical results, many clinical trials fail to demonstrate the benefits of a new pharmacological agent due to the absence of anticancer activity in cancer patients or the presence of toxicities incompatible with the continuation of the trial. The development of new clinical trials must consider the intra- and inter-tumor heterogeneity of CRC patients, which influences their responses to therapies. Nowadays, targeted therapies and immunotherapy have significantly improved the survival of CRC patients, and the newly developed therapies are increasing the therapeutic options for patients with advanced CRC harboring specific genetic abnormalities [5]. However, despite the initial success of commonly used therapies, most drugs fail to target the MRD associated with CSCs which often leads to relapse in cancer patients. Unfortunately, up to 50% of patients with early-stage CRC at diagnosis will eventually develop metastatic disease, and most of them have unresectable metastatic disease because of the size, location, and/or extent of the disease [76,77]. New clinical trials must therefore be designed to test drugs that could become relevant treatment options for patients with early-stage and advanced CRC. However, the lack of accurate preclinical models that take into account intrinsic and extrinsic characteristic of tumors, such as CSC subpopulation, tumor stroma and TME, is a major technical problem [134]. The CCSC isolation and characterization methods presented in this review highlighted the limitations of the methods currently in use, particularly those using CCSC markers. Cell sorting using phenotypic characteristics allows the sorting of only part of the CCSC population because they are heterogeneous, plastic, and subject to many signals from the TME. Thus, the use of new innovative techniques such as SdFFF which sorts cells according to cell characteristics other than marker expression or the combination of several isolation techniques is crucial. In conclusion, more accurate preclinical models are required because current approaches are not precise enough to identify therapies that may be clinically effective, particularly those targeting CCSCs [145].

## 6. Conclusions

Targeting CCSCs holds promise for preventing disease relapse and metastasis in CRC patients. In addition, as a major driving force of drug resistance, CCSCs are attractive potential targets for the treatment of CRC. However, the development of therapeutic agents specifically targeting CCSCs is complex, as highlighted by the clinical trial results presented in this review. Despite the increasing number of therapies, resistance mechanisms may emerge and thus complicate the therapeutic management of patients with CRC. In order to achieve a short- and long-term therapeutic response, the ideal therapeutic strategy should target both the cancer cells of the tumor mass to obtain tumor regression, CCSCs to prevent relapse and metastasis, and TME to limit cellular plasticity and the reappearance of CCSCs.

## Figures and Tables

**Table 1:** Experimental models, markers and CCSC isolation and characterization methods used in the first publications identifying CSCs in CRC.

References	Experimental Models	Identified CCSC Subpopulations	CCSC Isolation Methods	CCSC CharacterizationAssays
O’Brien et al. [20]	CRC patient tissues CRC cells from patient tumors Animal model (mice)	CD133^+^	MACS and FACS	Flow cytometry Immunohistochemistry Tumorigenicity assay
Ricci-Vitiani et al. [19]	CRC patient tissues CRC cells from patient tumors Primary tumor cell cultures Animal model (mice)	CD133^+^	MACS and FACS	Sphere formation assay Flow cytometry Immunohistochemistry Tumorigenicity assay
Dalerba et al. [22]	CRC patient tissues CRC xenograft lines Single-cell suspensions	EpCAM^high^/CD44^+^ EpCAM^high^/CD44^+^/CD166^+^	FACS	ALDH assay Flow cytometry Tumorigenicity assay
Barker et al. [23]	Animal model (Ah-cre/Apc^flox/flox^ and Lgr5-EGFP-IRES-creER^T2^/APC^flox/flox^ mice)	Lgr5^+^	/	LacZ analysis Immunohistochemistry
Sangiorgi and Capecchi [24]	Animal model (Bmi1-IRES-Cre-ER mice)	Bmi1^+^	/	LacZ analysis Immunohistochemistry
Vermeulen et al. [25]	CRC patient tissues CRC cells and single-cell-derived cultures from patient tumors Animal model (mice)	CD133^+^/CD24^+^ CD44^+^/CD166^+^ CD24^+^/CD29^+^	MACS and FACS	Sphere formation assay In vitro differentiation assay Immunohistochemistry Flow cytometry Tumorigenicity assay
Pang et al. [26]	CRC patient tissues CRC cells from patient tumors Animal model (mice)	CD133^+^/CD26^+^ CD133^+^/CD26^+^/CD44^+^	MACS and FACS	Sphere formation assay In vitro invasion assays Chemotherapeutic treatments Tumorigenicity assay
Todaro et al. [27]	CRC patient tissues Sphere-derived adherent cultures CRC cells from patient tumors or spheres Animal model (mice)	CD44v6^+^	MACS and FACS	Immunofluorescence Immunohistochemistry Invasion assay Sphere formation assay Tumorigenicity assay

CRC: colorectal cancer; CCSC: colorectal cancer stem cells; CD: cluster of differentiation; MACS: magnetic-activated cell sorting; FACS: fluorescence-activated cell sorting; ALDH: aldehyde dehydrogenase.

**Table 2:** Functions and roles in CRC prognosis of CCSC markers.

CCSC Markers	Functions	Roles in Prognosis of CRC	References
Bmi-1	Polycomb-repressor protein Involved in self-renewal	High expression of Bmi-1 is associated with poor survival	[23,24,31,32]
CD24 (Heat stable antigen 24)	Cell adhesion molecule Alternative ligand of P-selectin	Strong cytoplasmic expression of CD24 is correlated with shortened patient survival	[25,33]
CD26	Cell adhesion glycoprotein Promote invasion and metastases	Elevated-CD26 expression is associated with advanced tumor staging and worse overall survival	[26,34]
CD29 (Integrin-β1)	Transmembrane proteinInvolved in cell adhesion	Overexpression of CD29 is correlated with poor prognosis and aggressive clinicopathological features	[25,35]
CD44	Transmembrane glycoprotein Regulate cell interactions, adhesion and migration	CD44 overexpression is associated with lymph node metastasis, distant metastases and poor prognosis	[36,37,38]
CD44v6	Bind hepatocyte growth factor Promote migration and metastases	High level of CD44v6 has an unfavorable impact on overall survival	[27,29,38]
CD133 (Prominin-1)	Cell surface glycoprotein Regulate self-renewal and tumor angiogenesis	CD133 expression is correlated with low survival in CRC patients	[21,39,40]
CD166 (Activated leukocyte adhesion molecule)	Cell adhesion molecule Mediate homophilic interactions	Overexpression of CD166 is correlated with shortened patient survival	[22,25,41]
EpCAM (Epithelial cell adhesion molecule)	Transmembrane glycoprotein Regulate cell adhesion, proliferation and migration	Loss of EpCAM expression is associated with tumor stage, lymph node and distant metastases and poor prognosis	[22,37,42]
Lgr5 (Leucine-rich repeat- containing G-protein coupled receptor 5)	Seven-transmembrane protein Target of Wnt pathway involved in self-renewal	Lgr5 expression is associated with lymph node and distant metastases, and overexpression with reduced overall survival	[23,28,37,43]

CCSC: colorectal cancer stem cells; CD: cluster of differentiation; ECM: extracellular matrix; CRC: colorectal cancer.

**Table 3:** Advantages and disadvantages of CCSC isolation methods.

Features	Isolation Methods	Advantages	Disadvantages	References
Phenotypic	MACS	High specificity Fast and easy method	No universal CCSC marker Monoparameter separation	[18,31,32]
	FACS	High specificity Multiparameter separation	No universal CCSC marker Require large number of cells	[18,31]
Functional	ALDH activity assay	High stability	Low specificity	[47,48]
	Side population assay	No cell labelling required	Low purity and specificity	[49]
	Colony and sphere formation assay	No need for complicated laboratory equipment	Absence of standardized protocol Require proper cell dilution	[50,52,53]
	Tumorigenicity assay	Gold standard method	Complicated laboratory equipment Ethical consideration	[56,58]
Biophysical	SdFFF	No cell labelling required Cell size and density separation	Time consuming	[16,46,59]

CCSC: colorectal cancer stem cell; MACS: magnetic-activated cell sorting; FACS: fluorescence-activated cell sorting; ALDH: aldehyde dehydrogenase; SdFFF: sedimentation field flow fractionation.

**Table 4:** Systemic therapies for localized and advanced colorectal cancer.

Systemic Therapies	Drug Names	Functions	Recommendations	References
Chemotherapy	5-Fluorouracil	Antimetabolite	Localized and advanced tumors	[82]
	Capecitabine	Antimetabolite		[72]
	Irinotecan	Topoisomerase inhibitor		[83]
	Oxaliplatin	Alkylating agent		[84]
	Trifluridine/ Tipiracil	Nucleoside analog/ TP inhibitor		[85]
Targeted therapy	Bevacizumab	mAb anti-VEGF-A	*KRAS/NRAS/BRAF* Mutated tumors	[86]
	Regorafenib	Multikinase inhibitor targeting e.g., VEGFR and BRAF		[87]
	Aflibercept	Recombinant fusion protein blocking VEGF-A/B		[88]
	Ramucirumab	mAb anti-VEGFR-2		[89]
	Cetuximab	mAb anti-EGFR	*KRAS/NRAS/BRAF* Wild-type tumors	[90]
	Panitumumab			[90]
Immunotherapy	Pembrolizumab	mAb anti-PD-1	MSI-high tumors	[91]
	Nivolumab			[92]
	Ipilimumab	mAb anti-CTLA4		[92]
Newly developed therapy	Vemurafenib	BRAF inhibitors	*BRAF* V600E mutated tumors	[93]
	Dabrafenib			[93]
	Encorafenib			[94]
	Trametinib	MEK inhibitors		[93]
	Binimetinib			[94]
	Trastuzumab	mAb anti-HER2	*HER2* amplified tumors	[95]
	Pertuzumab			[95]
	Lapatinib	Dual HER2/EGFR inhibitor		[96]
	Larotrectinib	TRK inhibitors	*NTRK* gene fusion-positive tumors	[97]
	Entrectinib			[98]

TP: thymidine phosphorylase; mAb: monoclonal antibody; VEGF: vascular endothelial growth factor; VEGFR: vascular endothelial growth factor receptor; EGFR: epidermal growth factor receptor; PD-1: programmed death cell receptor 1; CTLA4: cytotoxic T-lymphocyte-associated antigen 4; MEK: mitogen-activated kinases; TRK: tropomyosin receptor kinases; MSI: microsatellite instability; NTRK: neurotrophic receptor tyrosine kinase gene.

**Table 5:** Clinical trials on colorectal cancer and cancer stem cells from ClinicalTrials.gov.

Trial Registration and Status	Study Titles	Interventions	Phases	Investigators
**NCT02753127** Active, not recruiting	A Study of Napabucasin (BBI-608) in Combination with FOLFIRI in Adult Patients with Previously Treated Metastatic Colorectal Cancer (CanStem303C)	Drug: Napabucasin	Phase III	Sumitomo Dainippon Pharma Oncology, Inc
**NCT01189942** Completed	A Study of FOLFIRI Plus OMP-21M18 as 1st or 2nd-line Treatment in Subjects with Metastatic Colorectal Cancer	Drug: OMP-21M18	Phase I	Mereo BioPharma (OncoMed Pharmaceuticals, Inc.)
**NCT02859415** Recruiting	Continuous 24 h Intravenous Infusion of Mithramycin, an Inhibitor of Cancer Stem Cell Signaling, in People with Primary Thoracic Malignancies or Carcinomas, Sarcomas or Germ Cell Neoplasms with Pleuropulmonary Metastases	Drug: Mithramycin	Phase I and II	National Cancer Institute
